# Dental caries risk in patients with chronic airway diseases using dry powder inhalers: a retrospective cohort study

**DOI:** 10.1186/s12903-026-08583-1

**Published:** 2026-05-21

**Authors:** Jinhee Ha, June Hong Ahn, Jong Geol Jang, Minkyeong Cho, Minae Park, Sojeong Park, Ji Sung Lee, Yeong Cheol Cho, Seung Won Ra

**Affiliations:** 1 Department of Dentistry, Ulsan University Hospital, University of Ulsan College of Medicine, Ulsan, South Korea; 2 Division of Pulmonology and Allergy, Department of Internal Medicine, Yeungnam University Medical Center, College of Medicine, Yeungnam University, Daegu, South Korea; 3 Department of Pharmacy, Ulsan University Hospital, University of Ulsan College of Medicine, Ulsan, South Korea; 4 Department of Data Science, Hanmi Pharm. Co., Ltd., Seoul, Republic of Korea; 5 Clinical Research Center, Asan Institute for Life Sciences, Asan Medical Center, University of Ulsan College of Medicine, Seoul, Republic of Korea; 6 Division of Pulmonology, Department of Internal Medicine, Ulsan University Hospital, University of Ulsan College of Medicine, Ulsan, South Korea; 7 Department of Medical Science Convergence, Graduate School of Medical Science, Basic-Clinical Convergence Research Institute, University of Ulsan, Ulsan, Republic of Korea

**Keywords:** Dry powder inhaler, Dental caries, Chronic airway disease, Lactose monohydrate

## Abstract

**Objective:**

Inhalers, which are essential for chronic airway disease treatment, differ in formulations. Dry powder inhalers (DPIs) with lactose monohydrate may increase the risk of dental caries compared with lactose-free pressurized metered-dose inhalers (pMDIs). We investigated the incidence of dental caries among users of these inhaler types for chronic airway diseases.

**Methods:**

In this study, we analyzed inhalation technique checklists and patient records at a single center from 2010 to 2018. The study included adult patients with asthma or chronic obstructive pulmonary disease who initiated inhaler usage for the first time and continued for at least 6 months. Those with preexisting dental caries, inhaler changes, or missing lung function data were excluded. We analyzed the incidence rate of dental caries during the study period, the time to the first dental caries occurrence, and the adjusted hazard ratios among pMDI and DPI users employing Kaplan–Meier survival analysis and Cox proportional hazards model.

**Results:**

Of 4,092 patients, 1,381 (33.7%) and 2,711 (66.3%) used pMDIs and DPIs, respectively. The incidence rate of dental caries was 2.28 per 1,000 person-years for pMDI users and 4.53 for DPI users. DPI users exhibited a significantly shorter time to first dental caries occurrence (*P* = 0.029, log-rank test) with a higher 5-year caries occurrence rate (1.9% vs. 0.8%, *P* = 0.015) and a higher risk of dental caries than pMDI users, with an adjusted hazard ratio of 2.09 (95% confidence interval: 1.04–4.18, *P* = 0.037) in the multivariable Cox regression analysis.

**Conclusions:**

Patients with chronic airway disease using DPIs have a higher risk of dental caries than those using pMDIs. Thus, vigilant dental health monitoring is necessary for DPI users.

**Supplementary Information:**

The online version contains supplementary material available at 10.1186/s12903-026-08583-1.

## Introduction

Chronic airway diseases, such as asthma and chronic obstructive pulmonary disease (COPD), are commonly managed with inhalation therapy [1, 2]. Inhalers directly deliver medication to the lungs and are mainly categorized into four types: nebulizers, soft mist inhalers (SMIs), pressurized metered-dose inhalers (pMDIs), and dry powder inhalers (DPIs) [3]. While nebulizers, SMIs, and pMDIs release an aerosol of medication inhaled by patients, DPIs contain medication in a dry powder form that is inhaled by patients. Lactose monohydrate is often utilized as a carrier for the active drug in DPI formulations; however, its presence in the oral cavity has raised concerns regarding its potential to contribute to dental caries, also known as tooth decay [4]. In addition to lactose exposure, inhaled medications may alter the oral environment by reducing salivary flow and buffering capacity, as well as lowering salivary pH. In particular, previous studies have suggested that prolonged use of β₂-agonists is associated with impaired salivary secretion, indicating that reduced salivary flow may be related not only to the underlying respiratory disease but also to the pharmacologic effects of the medication itself [5, 6]. Long-acting muscarinic antagonists, including tiotropium, glycopyrronium, aclidinium, and umeclidinium, are also associated with oral dryness because of their anticholinergic effects [7–9]. Collectively, these changes can impair mechanical cleansing, promote enamel demineralization, and facilitate the growth of acidogenic bacteria, thereby increasing susceptibility to dental caries. Dental caries is a multifactorial disease caused by the interaction among sugar, bacteria, and host factors, leading to the demineralization of the tooth enamel and dentin; the risk of dental caries is influenced by various factors, including oral hygiene practices, dietary habits, salivary flow, and exposure to fluoride [10, 11]. Despite the widespread use of inhalers, research on the association between inhaler type and dental caries development in adult patients with chronic airway diseases is scarce. Considering the potential implications for oral health, it is imperative to understand whether patients using DPIs have a higher incidence of dental caries than those using pMDIs, which do not contain lactose monohydrate. DPIs and pMDIs differ in their physical and chemical formulations as well as in their aerosol delivery mechanisms. DPIs deliver medication in a dry powder form composed of micronized drug particles that are commonly blended with carrier particles such as lactose monohydrate to improve powder flowability, dispersion, and dose uniformity [12]. In contrast, pMDIs deliver aerosolized medication using a propellant-based system in which the drug is suspended or dissolved in a liquefied propellant, typically hydrofluoroalkane, without lactose excipients. These formulation differences lead to distinct aerosolization mechanisms. The performance of DPIs depends largely on the patient’s inspiratory flow to disperse and deagglomerate powder particles, whereas pMDIs generate aerosol droplets through propellant expansion and device actuation [13]. Consequently, particle size distribution, aerosol velocity, and patterns of drug deposition in the oropharyngeal cavity may differ between the two inhaler types. In DPIs, the relatively low initial velocity of powder particles and the presence of lactose carrier particles may increase the likelihood that residual powder deposits on oral mucosal or dental surfaces [14]. Furthermore, lactose-containing DPI formulations have been reported to exhibit lower pH values and greater titratable acidity than non-lactose inhaler formulations, potentially contributing to a more acidic oral environment [15]. In contrast, pMDIs typically produce high-velocity aerosol sprays that may allow a greater proportion of drug particles to pass through the oral cavity and reach the lower airways [13]. These formulation and aerosolization differences may influence drug deposition patterns in the oral cavity, as powder particles from DPIs may adhere more readily to mucosal and dental surfaces. Considering these differences, it is important to determine whether patients using DPIs have a higher incidence of dental caries than those using pMDIs.

The excipients in DPIs contain lactose monohydrate, which acts as a drug carrier and has been shown to exert cariogenic effects when salivary flow is reduced [16]. The lactose content significantly varies between products; for instance, Seretide Diskus^®^ contains 12.5 mg per dose, whereas Symbicort Turbuhaler^®^ contains 0.73 mg per dose, representing more than a 12-fold difference [17].

Previous studies investigating the association between asthma and dental caries risk have yielded inconsistent results [18–20]. We hypothesize that these conflicting findings may be attributed to variations in inhaler formulations. Specifically, DPI, which contains lactose monohydrate as a carrier, may increase the risk of dental caries development compared with pMDI, which does not contain this potentially cariogenic component. This study aimed to compare the incidence of dental caries between adult patients with chronic airway disease using DPIs and those using pMDIs.

## Materials and methods

### Study design and subjects

**Fig. 1:**
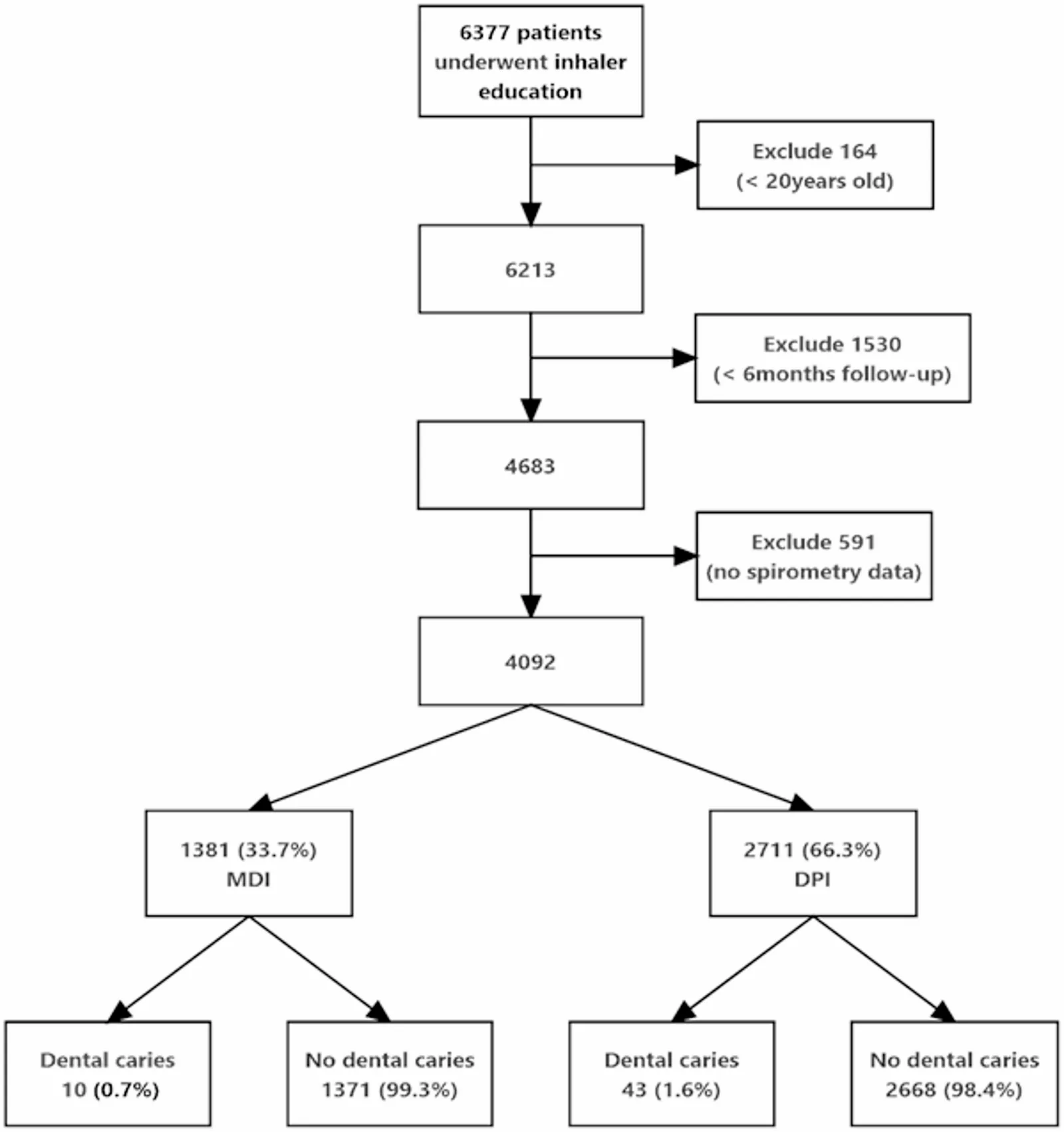
Flowchart of the study

This study was conducted in Ulsan University Hospital between January 2010 and October 2018. It enrolled adult patients with asthma or COPD who used an inhaler for the first time and participated in the training sessions for inhalation technique. Patients initiating inhaler therapy received education from a clinical pharmacist using a standardized inhaler education checklist. Separate 10-step checklists were used for pressurized metered-dose inhalers (pMDIs) and dry powder inhalers (DPIs) to assess inhaler technique and patient understanding. Examples of the counseling record and inhaler technique checklists used in this study are presented in Supplementary Table 1. Although post-inhalation oral hygiene practices, such as mouth rinsing after inhaler use, were not individually documented for retrospective analysis, all consecutively enrolled first-time inhaler users received standardized inhaler education by a clinical pharmacist at the initiation of inhaler therapy, including instruction on mouth rinsing after inhaler use. We analyzed the data from inhalation technique checklists and electronic medical records. Of the 6,377 participants, those aged ≥ 20 years with permanent dentition and who had used any type of inhaler for more than 6 months due to the diagnosis of asthma or COPD were included. Patients with ICD-10 codes for dental caries within 1 year before the first day of the inhaler training session, those who had switched inhalers more than once, and those lacking spirometry data were excluded. Ultimately, a total of 4,092 patients were included in the final analysis (Fig. 1). Clinical and demographic data were extracted from electronic medical records. These included age, sex, smoking status, socioeconomic status, pulmonary function test results, and the number of inhaler education sessions. Pulmonary function parameters such as forced expiratory volume in one second (FEV_1_) were used to reflect the clinical severity of the underlying airway disease. However, detailed information on general signs and symptoms, individual adherence to inhaler technique after education, and systemic antibiotic use was not systematically recorded in a manner suitable for retrospective analysis.

The inhaler types in this study were pMDIs and DPIs (Supplementary Tables 2 and 3). Inhaler devices that do not contain lactose monohydrate as a carrier, such as SMI, were categorized as pMDIs. All cases of dental caries in the study were diagnosed by the dentist at Ulsan University Hospital. Dental caries events were identified based on newly assigned ICD-10 diagnostic codes (K02) recorded in the electronic medical records. Clinical diagnosis was performed during routine dental examinations at the Department of Dentistry, Ulsan University Hospital by a board-certified specialist in conservative dentistry. Examinations were conducted under dental unit lighting, using visual-tactile inspection with radiographic evaluation when indicated. The incidence of dental caries was defined as the first occurrence of a newly recorded ICD-10 K02 code after the index date. Dental examinations were not performed at predefined study intervals; instead, patients visited the dental clinic when they developed oral symptoms or problems, and caries events were identified during those routine clinical visits. The patients’ medical records, including age, gender, body mass index (BMI), smoking status, socioeconomic status, number of inhaler education sessions, history of inhaler switch, and pulmonary function test results, were recorded.

### Statistical analyses

The main outcome was a new diagnosis of dental caries during follow-up. Dental caries-free survival was evaluated using Kaplan–Meier curves according to the inhaler type used (DPI vs. non-DPI), and the groups were compared using the log-rank test. A Cox proportional hazards regression analysis with adjustments for age, sex, smoking status, and FEV_1_ was conducted to determine the independent association between DPI use and dental caries risk. Continuous variables were expressed as mean ± standard deviation and compared using Student’s *t*-test, whereas categorical variables were compared using the chi-squared test. The incidence rates were calculated by dividing the occurrence rate of dental caries by the person-years of follow-up. Dental caries-associated variables with a *P-*value < 0.1 in the univariate analyses were further examined in the multivariate analyses to determine the hazard ratios (HRs) and 95% confidence intervals (CIs). Age, sex, smoking status, and FEV_1_ were also included in the Cox proportional hazards regression analysis. In all analyses, a two-tailed *P-*value < 0.05 was considered to indicate statistical significance. All statistical analyses were conducted using the SPSS software (ver. 24.0; SPSS Inc., Chicago, IL, USA).

## Results

### Clinical characteristics

Of the 4,092 included patients, 1,381 (33.7%) and 2,711 (66.3%) were pMDI and DPI users, respectively. In the pMDI group, 10 (0.7%) developed dental caries, whereas in the DPI group, 43 (1.6%) developed dental caries. Baseline characteristics are summarized in Table 1. The DPI group showed a significantly higher proportion of men, a lower proportion of never-smokers, and significantly lower pulmonary function parameters (FEV_1_ and FEV_1_/FVC) compared with the pMDI group (*p* < 0.001 for all comparisons). No significant difference in BMI, socioeconomic status, FVC, number of inhaler education sessions, and history of inhaler switch was observed between the groups.

**Table 1:** Baseline characteristics of the study subjects according to inhaler type

	pMDI *n* = 1,381	DPI *n* = 2,711	*p*-value^*^
Age, years	62.8 ± 14.7	62.7 ± 13.6	0.708
Gender, men	690 (50.0%)	1,596 (58.9%)	< 0.001
BMI, kg/m^2^	24.0 ± 4.0	24.0 ± 3.9	0.692
Smoking status	*n* = 1,380	*n* = 2,709	< 0.001
Never-smoker	677 (49.1%)	1,048 (38.7%)	
Ever-smoker	703 (50.9%)	1,661 (61.3%)	
Current smoker, pack-years	*n* = 200, 34.6 ± 21.9	*n* = 455, 34.6 ± 19.7	0.988
Socioeconomic status	*n* = 1,371	*n* = 2,668	0.321
Ordinary	1,280 (93.4%)	2,512 (94.2%)	
Poor	91 (6.6%)	156 (5.8%)	
FEV_1_, % predicted	73.1 ± 23.3	70.2 ± 22.2	< 0.001
FVC, % predicted	82.1 ± 19.5	82.8 ± 17.5	0.250
FEV_1_/FVC %	66.0 ± 15.2	62.7 ± 16.0	< 0.001
Number of education sessions	1.43 ± 0.84	1.44 ± 0.84	0.652
Any inhaler switch	166 (12.0%)	321 (11.8%)	0.867
MDI to DPI switch or vice versa	101 (7.3%)	179 (6.6%)	0.394

Data are expressed as mean ± standard deviation or absolute number (%)

*MDI *metered-dose inhaler, *DPI *dry powder inhaler, *BMI *body mass index, *FEV*_*1*_ forced expiratory volume in one second, *FVC *forced vital capacity

*Student’s t-test or chi-squared test

### Incidence rate and proportion of dental caries according to inhaler type

**Fig. 2:**
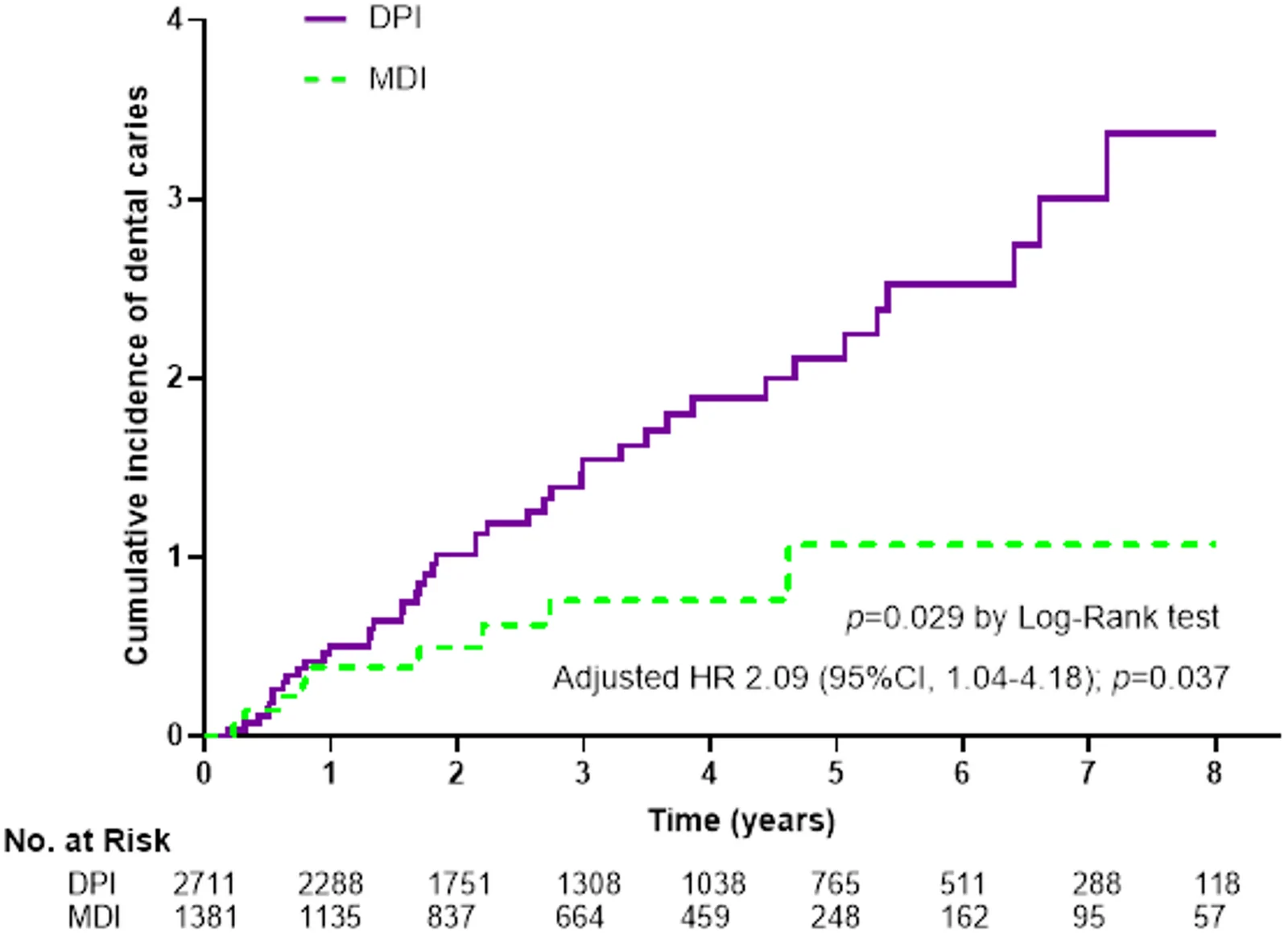
Proportion of dental caries by inhaler type

Table 2 presents the incidence rate of dental caries along with the unadjusted and adjusted HRs from the Cox proportional hazards regression analyses, with the pMDI group as the reference. The incidence rate of dental caries was 2.28 for the pMDI group vs. 4.53 for the DPI group per 1,000 person-years. In addition, the DPI group exhibited a significantly shorter time to dental caries occurrence than the pMDI group (*P* = 0.029 by the log-rank test) (Fig. 2) and a higher 5-year caries occurrence rate (1.9% vs. 0.8%, *P* = 0.015). In multivariable regression analyses, the DPI group exhibited significantly increased dental caries risk compared with the pMDI group after adjustment for age, sex, smoking status, and FEV_1_ (adjusted HR, 2.09; 95% CI, 1.04–4.18, *P* = 0.037).

**Table 2:** Incidence rate of dental caries and hazard ratios from the Cox regression analysis

					Unadjusted model		Multivariable	
Inhaler type	No. of patients	Events (*n*)	Follow-up duration (person-years)	Incidence rate per 1,000 person-years(95% CI)	HR (95% CI)	*P*-value	HR (95% CI)	*P*-value^*^
pMDI	1,381	10	4,379	2.28 (1.23–4.24)	1 (Ref.)		1 (Ref.)	
DPI	2,711	43	9,489	4.53 (3.36–6.11)	2.01 (1.01–4.01)	0.047	2.09 (1.04–4.18)	0.037

*CI *confidence interval, *DPI *dry powder inhaler, *FEV*_*1*_ forced expiratory volume in one second, *HR *hazard ratio, *pMDI *pressurized metered-dose inhaler

*Adjusted for age, sex, smoking status, and FEV_1_

The occurrence rate of dental caries was higher in the DPI group (1.1% vs. 0.4%, *P* = 0.063) compared with the pMDI group when only inhalers with the same active pharmaceutical ingredient were considered (Table 3).

**Table 3:** Occurrence rate of dental caries by inhaler type with the same active pharmaceutical ingredient

	DPI	pMDI	*P*-value
Seretide Diskus vs. Evohaler (*n* = 975 vs. 262)	1.2%	0.8%	0.746
Symbicort Turbuhaler vs. Rapihaler (*n* = 1,011 vs. 205)	1.0%	0.5%	0.702
Spiriva HandiHaler vs. Respimat (*n* = 856 vs. 480)	1.1%	0.4%	0.346
Total	1.1%	0.4%	_0.063_

*DPI *dry powder inhaler, *pMDI *pressurized metered-dose inhaler

## Discussion

In this study, the incidence rate of dental caries in permanent dentition was evaluated in patients with chronic airway disease who participated in the training sessions for inhalation technique. The incidence rate of dental caries was 2.28 per 1,000 person-years for pMDI users and 4.53 per 1,000 person-years for DPI users. In the multivariable Cox regression analysis, the DPI group showed a twofold higher risk of dental caries than the pMDI group after adjustment for age, sex, smoking status, and FEV_1_. To our knowledge, this is the first study to investigate the difference in the incidence rate of dental caries between the types of inhalers. Several studies have investigated the association between chronic airway disease per se and oral health and yielded inconsistent results. A cross-sectional study reported that subjects with a history of COPD had more periodontal attachment loss than those without [21]. Furthermore, a meta-analysis revealed that periodontal disease is a significant and independent risk factor of COPD [22]. Another meta-analysis revealed that individuals with asthma are nearly twice as likely to develop dental caries compared with those without asthma, with asthma medication use identified as an important source of variation in the meta-regression analysis [19]. However, the causal link between inhaler use and dental caries in patients with chronic airway diseases remains unclear, indicating the need for further research into the underlying mechanisms, particularly the influence of asthma medications. Some studies have reported that inhalers used for childhood asthma were not associated with an increased risk of dental caries [23] and the prevalence of dental caries did not significantly differ between children with asthma and healthy controls [24–26]. Notably, these studies did not specify the types of inhalers used nor did they compare the caries risk between pMDIs and DPIs. Children with asthma, particularly those below 4–5 years old, often lack the inspiratory capacity needed to use DPIs, leading to the predominant prescription of pMDIs in clinical practice. This reliance on non-lactose-based pMDIs in young children could explain the lack of association between asthma, inhaler use, and caries risk in these studies.

In DPIs, fine drug particles are blended with larger carrier particles in a dry powder mixture. These carrier particles improve the handling, dispensing, and metering of the drug. The main excipient used in DPIs is lactose monohydrate [16, 27]. Among all sugars, lactose is considered to be the least cariogenic, but it has been proven to cause demineralization of the tooth enamel and root dentin, causing tooth decay [16]. Lactose-based DPIs exhibit a significantly lower inherent pH than MDIs and a higher titratable acidity than non-lactose DPIs, resulting in significant drops in salivary pH up to 20 min after use [15]. The potential cariogenicity of lactose-based DPIs should be taken into consideration.

Inhaler medications may also contribute to dental caries. Long-acting bronchodilators include long-acting β₂-agonists (LABA) and long-acting muscarinic antagonists. Prolonged use of LABA has been suggested to be associated with diminished salivary production and secretion and thus increased frequency of dental caries [5]. The flow rates of whole and parotid saliva are decreased by 26% and 36%, respectively. Furthermore, an acidic environment is induced and persists due to the loss of the salivary buffering capacity. This in turn promotes the growth of acidic bacteria, such as *Lactobacillus* and *Streptococcus mutans*, which grow under acidic conditions and continue to metabolize carbohydrates in low-pH environments. This leads to uncontrolled carious attack [28]. Inhaled corticosteroids also play a pivotal role in the management of asthma and severe COPD. Inhaled corticosteroids are weak organic acids and are generally not metabolized by oral bacteria. However, they pose a threat to pH when sugar-based inhalers are used. Because all the patients in the study group were using lactose-based DPIs, their cariogenicity in addition to the medication effects could not be neglected. Furthermore, as the lactose content varies across different medications, the manifestation of dental caries can differ accordingly.

Inhalation techniques can also theoretically affect drug deposition in the oral cavity and be associated with dental caries. Marzie et al. reported no significant correlation between inhalation technique and dental caries in patients with asthma [29]. However, inhalation technique was relatively acceptable in all patients with asthma in that study. Moreover, the majority of studies presented results without distinguishing between MDIs and DPIs. Therefore, the potential differences in dental caries induction between the two medications could not be confirmed. Providing education on the correct use of inhalers, encouraging regular dental check-ups, and promoting oral hygiene practices, including tooth brushing, are the recommended prophylactic strategies for patients using inhalers. In our institution, patients who are prescribed inhaler therapy routinely receive standardized education from a specialized clinical pharmacist regarding the correct inhaler technique as well as oral care after inhaler use. This educational program may contribute to reducing variability in oral hygiene behaviors among inhaler users. In addition, the study population was not selectively recruited; rather, patients were consecutively included at the initiation of inhaler therapy. Dental caries events were subsequently identified during routine visits to the dental department when patients presented with oral symptoms or problems, which may have helped reduce potential selection bias in outcome ascertainment.

This study has several limitations. First, the diagnosis of dental caries may be underdiagnosed or missed in some study participants. All cases of dental caries were diagnosed solely by dentists at Ulsan University Hospital, and no data were available on diagnoses from other healthcare institutions. Missing data due to loss to follow-up among patients referred from the department of internal medicine to the department of dentistry may have affected the results of this study. However, most of the patients with dental problems while using inhalers in this study were likely to have been referred to Ulsan University Hospital by their respiratory physician. Second, there may be heterogeneity in the association between dental caries and COPD or asthma, but this was not separately analyzed in this study. However, the results are meaningful because they were corrected for age, sex, smoking status, and FEV_1_ in the Cox regression analysis, which may be different in the two diseases. Third, the dose of lactose monohydrate varies for each DPI type, and the dose of DPI varies for each patient, but a more specific analysis on this aspect has not been conducted. Lastly, data on other factors contributing to tooth decay, such as comorbidities and critical errors in inhaler use, were limited. While personal oral hygiene practices, such as nighttime brushing and the use of dental floss or interdental brushes, are likely to be strongly linked to the incidence of dental caries, these factors were not comprehensively examined in this study. In addition, information regarding inhaler technique and post-inhalation oral hygiene practices, such as mouth rinsing, was not available in the medical records. Dental caries is a multifactorial disease influenced by oral hygiene behaviors, dietary habits such as sugar intake, and socioeconomic factors. Therefore, residual confounding cannot be completely excluded in this retrospective analysis. Although we adjusted for age, sex, smoking status, and pulmonary function, matching based on behavioral variables was not feasible due to limitations in the available clinical data. Additionally, in this study, periodontal disease was not evaluated through radiographic examinations or periodontal assessments. Further large-scale studies using health insurance data are warranted. There is also a need to develop lactose-free dry powder inhaler formulations with lower cariogenic potential. Although these limitations may influence the accuracy and generalizability of the findings, this study provides preliminary evidence suggesting an association between DPI use and dental caries in patients with chronic airway disease. Future prospective studies with more comprehensive clinical assessments and detailed evaluation of oral health behaviors are warranted to further validate these findings. Long-term follow-up studies stratified by inhaler formulation and dosage may help clarify potential dose-response relationships. In addition, large-scale population-based analyses using linked health insurance and oral health examination data may provide broader insight into the long-term oral health impact of inhaler therapies.

In conclusion, the use of DPIs in patients with chronic airway disease was associated with a significantly higher risk of dental caries than the use of pMDIs. Moreover, the time to first dental caries occurrence was shorter in the DPI group. For patients with chronic airway disease using DPIs, preventive oral care, regular dental assessment, and timely treatment of dental caries are warranted.

## Supplementary Information

Supplementary Material 1.

Supplementary Material 2.

Supplementary Material 3.

## Data Availability

The datasets used and/or analyzed during the current study are available from the corresponding author on reasonable request.

## Abbreviations

DPI: Dry powder inhaler
pMDI: Pressurized metered-dose inhaler
COPD: Chronic obstructive pulmonary disease
FEV1: Forced expiratory volume in one second
FVC: Forced vital capacity
BMI: Body mass index
ICD-10: International Classification of Diseases, 10th Revision
HR: Hazard ratio
CI: Confidence interval
