# Supplementary material for: Dental caries risk in patients with chronic airway diseases using dry powder inhalers: a retrospective cohort study
Source: BMC Oral Health. 2026 May 21;26:1595. doi: 10.1186/s12903-026-08583-1 (PMC13508197; doi:10.1186/s12903-026-08583-1)
Supplement: Supplementary file 1 [file 12903_2026_8583_MOESM1_ESM.docx]

Supplementary Table 1

Standardized inhaler technique checklist for pressurized metered-dose inhalers (pMDIs)

| step | Inhaler technique assessment |
| --- | --- |
| 1 | Hold the inhaler correctly |
| 2 | Shake the inhaler 2–3 times before use |
| 3 | Breathe out fully |
| 4 | Tilt the head back slightly |
| 5 | Place the mouthpiece in the mouth |
| 6 | Inhale slowly |
| 7 | Hold the breath for 10 seconds |
| 8 | Breathe out through the nose |
| 9 | Wait before the next inhalation |
| 10 | Rinse the mouth after inhalation |

Standardized inhaler technique checklist for dry powder inhalers (DPIs)

| STEP | Inhaler technique assessment |
| --- | --- |
| 1 | Hold the inhaler correctly |
| 2 | Load the medication dose |
| 3 | Breathe out fully |
| 4 | Tilt the head back slightly |
| 5 | Place the mouthpiece in the mouth |
| 6 | Inhale deeply and forcefully |
| 7 | Hold the breath for 10 seconds |
| 8 | Breathe out through the nose |
| 9 | Wait before the next inhalation |
| 10 | Rinse the mouth after inhalation |
