# Supplementary material for: Dental caries risk in patients with chronic airway diseases using dry powder inhalers: a retrospective cohort study
Source: BMC Oral Health. 2026 May 21;26:1595. doi: 10.1186/s12903-026-08583-1 (PMC13508197; doi:10.1186/s12903-026-08583-1)
Supplement: Supplementary file 2 [file 12903_2026_8583_MOESM2_ESM.docx]

Supplementary Table 2. List of pMDIs included in the study

| Brand name | Ingredient | Pharmacologic class |
| --- | --- | --- |
| Alvesco Inhaler | Ciclesonide | ICS |
| Flutiform Inhaler | Fluticasone propionate / Formoterol fumarate dihydrate | ICS + LABA |
| Foster Inhaler | Beclomethasone dipropionate / Formoterol fumarate hydrate | ICS + LABA |
| Seretide Evohaler | Fluticasone propionate / Salmeterol xinafoate | ICS + LABA |
| Symbicort Rapihaler | Budesonide / Formoterol fumarate hydrate | ICS + LABA |
| Vahelva Respimat | Olodaterol hydrochloride / Tiotropium bromide hydrate | LABA + LAMA |
| Spiriva Respimat | Tiotropium bromide hydrate | LAMA |

pMDI, pressurized metered-dose inhaler; ICS, inhaled corticosteroid; LABA, long-acting β₂-agonist; LAMA, long-acting muscarinic antagonist.
