# Supplementary material for: Dental caries risk in patients with chronic airway diseases using dry powder inhalers: a retrospective cohort study
Source: BMC Oral Health. 2026 May 21;26:1595. doi: 10.1186/s12903-026-08583-1 (PMC13508197; doi:10.1186/s12903-026-08583-1)
Supplement: Supplementary file 3 [file 12903_2026_8583_MOESM3_ESM.docx]

Supplementary Table 3. List of DPIs included in the study

| Brand name | Ingredient | Pharmacologic class |
| --- | --- | --- |
| Flixotide Diskus | Fluticasone propionate | ICS |
| Pulmicort Turbuhaler | Budesonide | ICS |
| Relvar Ellipta | Fluticasone furoate / Vilanterol trifenatate | ICS + LABA |
| Seretide Diskus | Fluticasone propionate / Salmeterol xinafoate | ICS + LABA |
| Symbicort Turbuhaler | Budesonide / Formoterol fumarate dihydrate | ICS + LABA |
| Onbrez Breezhaler | Indacaterol maleate | LABA |
| Anoro Ellipta | Vilanterol trifenatate / Umeclidinium | LABA + LAMA |
| Duaklir Genuair | Formoterol fumarate dihydrate / Aclidinium bromide | LABA + LAMA |
| Xoterna Breezhaler | Indacaterol maleate / Glycopyrronium bromide | LABA + LAMA |
| Eklira Genuair | Aclidinium bromide | LAMA |
| Incruse Ellipta | Umeclidinium | LAMA |
| Spiriva HandiHaler | Tiotropium bromide hydrate | LAMA |

DPI, dry powder inhaler; ICS, inhaled corticosteroid; LABA, long-acting β₂-agonist; LAMA, long-acting muscarinic antagonist.
